# Development of Porous Coatings Enriched with Magnesium and Zinc Obtained by DC Plasma Electrolytic Oxidation

**DOI:** 10.3390/mi9070332

**Published:** 2018-07-02

**Authors:** Krzysztof Rokosz, Tadeusz Hryniewicz, Sofia Gaiaschi, Patrick Chapon, Steinar Raaen, Winfried Malorny, Dalibor Matýsek, Kornel Pietrzak

**Affiliations:** 1 Division of BioEngineering and Surface Electrochemistry, Department of Engineering and Informatics Systems, Koszalin University of Technology, Racławicka 15-17, PL 75-620 Koszalin, Poland; Tadeusz.Hryniewicz@tu.koszalin.pl (T.H.); kornel.pietrzak@s.tu.koszalin.pl (K.P.); 2 HORIBA France S.A.S., Avenue de la Vauve-Passage Jobin Yvon, 91120 Palaiseau, France; sofia.gaiaschi@horiba.com (S.G.); patrick.chapon@horiba.com (P.C.); 3 Department of Physics, Norwegian University of Science and Technology (NTNU), Realfagbygget, E3-124 Høgskoleringen 5, 7491 NO Trondheim, Norway; steinar.raaen@ntnu.no; 4 Faculty of Engineering, Hochschule Wismar-University of Applied Sciences Technology, Business and Design, DE 23966 Wismar, Germany; winfried.malorny@hs-wismar.de; 5 Institute of Geological Engineering, Faculty of Mining and Geology, VŠB—Technical University of Ostrava, 708 33 Ostrava, Czech Republic; dalibor.matysek@vsb.cz

**Keywords:** plasma electrolytic oxidation, micro arc oxidation, DC PEO, titanium, zinc nitrate hexahydrate Zn(NO_3_)_2_·6H_2_O, magnesium nitrate hexahydrate Mg(NO_3_)_2_·6H_2_O, 85% phosphoric acid H_3_PO_4_

## Abstract

Coatings with developed surface stereometry, being based on a porous system, may be obtained by plasma electrolytic oxidation, PEO (micro arc oxidation, MAO). In this paper, we present novel porous coatings, which may be used, e.g., in micromachine’s biocompatible sensors’ housing, obtained in electrolytes containing magnesium nitrate hexahydrate Mg(NO_3_)_2_·6H_2_O and/or zinc nitrate hexahydrate Zn(NO_3_)_2_·6H_2_O in concentrated phosphoric acid H_3_PO_4_ (85% *w*/*w*). Complementary techniques are used for coatings’ surface characterization, such as scanning electron microscopy (SEM), for surface imaging as well as for chemical semi-quantitative analysis via energy dispersive X-ray spectroscopy (EDS), X-ray photoelectron spectroscopy (XPS), glow discharge optical emission spectroscopy (GDOES), and X-ray powder diffraction (XRD). The results have shown that increasing contents of salts (here, 250 g/L Mg(NO_3_)_2_·6H_2_O and 250 g/L Zn(NO_3_)_2_·6H_2_O) in electrolyte result in increasing of Mg/P and Zn/P ratios, as well as coating thickness. It was also found that by increasing the PEO voltage, the Zn/P and Mg/P ratios increase as well. In addition, the analysis of XPS spectra revealed the existence in 10 nm top of coating magnesium (Mg^2+^), zinc (Zn^2+^), titanium (Ti^4+^), and phosphorus compounds (PO_4_^3−^, or HPO_4_^2−^, or H_2_PO_4_^−^, or P_2_O_7_^4−^).

## 1. Introduction

In the literature, the terms plasma electrolytic oxidation (PEO) or micro arc oxidation (MAO) refer to the electrochemical method of surface treatment of lightweight metals and their alloys, which leads to the spontaneous development of an oxide layer on their surfaces. These alloys usually consist of elements/metals, which may be found in the fourth and fifth B groups of the periodic table, i.e., titanium [1], zirconium [2,3], hafnium [4,5], niobium [6,7], tantalum [8,9], though the first works related to PEO (MAO) technique were carried out on aluminum, magnesium, and their alloys [10,11,12,13,14]. This method leads to locally strong nonlinear conditions of plasma discharges with high temperature, that results in the formation, on treated material, of a new porous coating [15] enriched with both electrolyte and substrate elements. It should also be noted that the use of the PEO (MAO) treatment creates coatings enriched with selected chemical elements in micro scale, while the nanolayers may be fabricated with the use of standard electropolishing [16], magnetoelectropolishing [17], or high current electropolishing [18]. Thicknesses of the coatings vary from 1 µm up to hundreds of micrometers [7,19,20], which results in different properties of the surface coating/passive layer compared to the metallic matrix. These phenomena are used in a variety of applications, including catalysts [21], biomedical implantable devices, joint prostheses, fracture fixation devices and dental implants [22], aerospace [23], chemical sensors [24], and wear-resistant materials [25]. The chemical composition, corrosion resistance of PEO coatings, as well as their thickness and porosity, depend on the electrolyte composition and used voltage or current regime (DC, AC, pulse). In addition, the frequency and shape of the voltage have influence on the properties of the obtained PEO coatings. As reported by Yong Han and Kewei Xu, amorphous titanium coatings may be obtained in electrolyte containing dicalcium phosphate CaHPO_4_, while the nanocrystallized structures may be formed in electrolytic solutions containing sodium carbonate Na_2_CO_3_ and acetate monohydrate (CH_3_COO)_2_Ca [26]. To fabricate the porous PEO coatings containing titanium oxides (anatase and/or apatite and/or rutile), a voltage from 250 V up to 450 V with pulse resume fixed at 100 Hz (duty cycle equaling from 3% [27]) up to 20% [28] or voltage of 250–500 V with pulse frequency of 1000 Hz (duty circle equals to 60%) [29,30] in aqueous electrolytes containing acetate monohydrate (CH_3_COO)_2_Ca and β-glycerophosphate disodium salt pentahydrate C_3_H_7_Na_2_O_6_P, can be used. The team of Long-Hao Li, Young-Min Kong at al. [31] published information that the use of DC voltage lower than 250 V, i.e., 190–600 V, with frequency 660 Hz (duty cycle equaling from 10%) in aqueous electrolyte containing of calcium acetate monohydrate (CH_3_COO)_2_Ca·H_2_O and calcium glycerophosphate CaC_3_H_7_O_6_P [32] may be used to create the porous PEO coatings of titanium oxides. It is also reported that, for PEO oxidation, the other electrolytes and voltages listed in Table 1, were used.

Based on the chemical composition of electrolytes reported in literature, the authors decided to propose a new electrolyte containing magnesium (Mg^2+^), zinc (Zn^2+^), and phosphate PO_4_^3−^ ions. Previous authors’ studies [47,48,49,50] clearly indicate that it is possible to obtain the porous coatings by plasma electrolytic oxidation on titanium [47,48] and its alloys [49,50] in electrolytes based on concentrated orthophosphoric acid with selected nitrates. The conducted research indicates that the porosity is gained in an entire volume of the obtained coatings, which are enriched with elements originating mainly from the electrolyte. In the present paper, we will present porous coatings obtained at voltages in the range of 450–650 V using new solutions, and their characterization by complementary techniques. Such results could lead to establishing a novel knowledge to be used in any micromachines’ applications.

## 2. Materials and Methods

Samples of commercially pure titanium grade 2, with dimensions of 10 × 10 × 2 mm, were used for plasma electrolytic oxidation (PEO) process, and then characterized by scanning electron microscope (SEM), energy dispersive X-ray Spectroscopy (EDS), X-ray powder diffraction (XRD), glow discharge optical emission spectroscopy (GDEOS), and X-ray photoelectron spectroscopy (XPS). In the first part of the studies (preliminary tests), the three-phase transformer with six diodes of Graetz bridge as the voltage source of 450 ± 46 V with pulsation frequency of 300 Hz was used, while during the second part (main studies), the PWR 1600 H (KIKUSUI ELECTRONICS CORP., Yokohama, Japan), Multi Range DC Power Supply 1600 W, 0–650 V/0–8 A as a power source of three voltages, i.e., 500 V, 575 V and 600 V, was used. All the PEO treatments were performed for 3 min in 500 mL of electrolytes containing magnesium nitrate hexahydrate Mg(NO_3_)_2_·6H_2_O and/or zinc nitrate hexahydrate Zn(NO_3_)_2_·6H_2_O in phosphoric acid (85% *w*/*w*). In details, all the solutions are presented in Table 2. The methodology of scanning electron microscopy (SEM), energy dispersive spectroscopy (EDS), glow discharge optical emission spectroscopy (GDOES), and X-ray photoelectron spectroscopy (XPS) [51,52,53,54,55] are presented in Table 3, and in detail, in the authors’ reference [48].

## 3. Results

**Figure 1:**
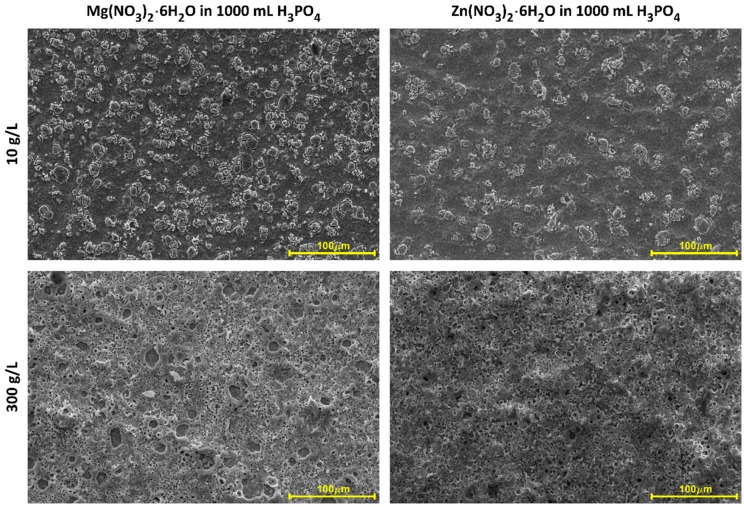

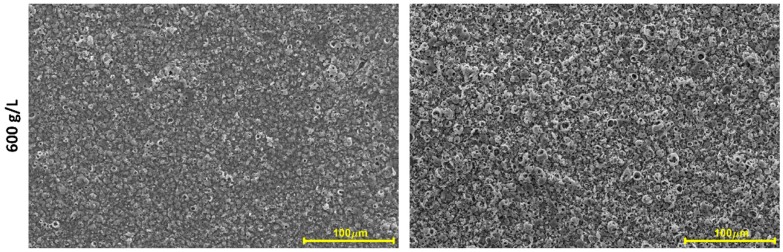
SEM micrographs of samples surfaces after PEO processing obtained in electrolytes composed of Mg(NO_3_)_2_·6H_2_O and Zn(NO_3_)_2_·6H_2_O in H_3_PO_4_ in three concentrations 10 g/L, 300 g/L, and 600 g/L, at a voltage of 450 ± 46 V, with a pulsation frequency of 300 Hz. Magnification 1000 times.

**Figure 2:**
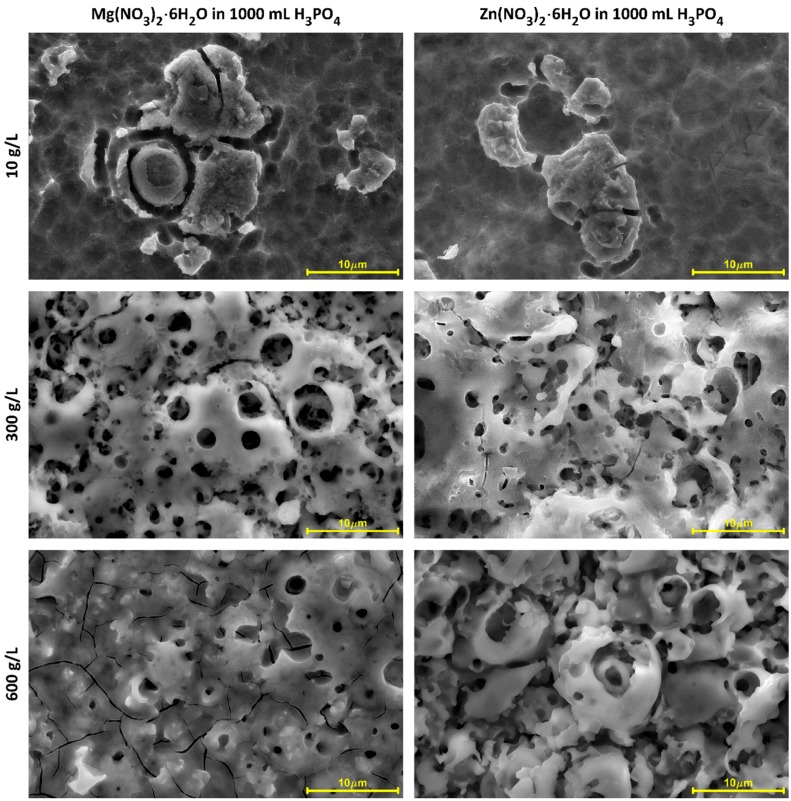
SEM micrographs of samples surfaces after PEO processing obtained in electrolytes composed of Mg(NO_3_)_2_·6H_2_O and Zn(NO_3_)_2_·6H_2_O in H_3_PO_4_ in three concentrations 10 g/L, 300 g/L, and 600 g/L, at a voltage of 450 ± 46 V, with a pulsation frequency of 300 Hz. Magnification 10,000 times.

**Figure 3:**
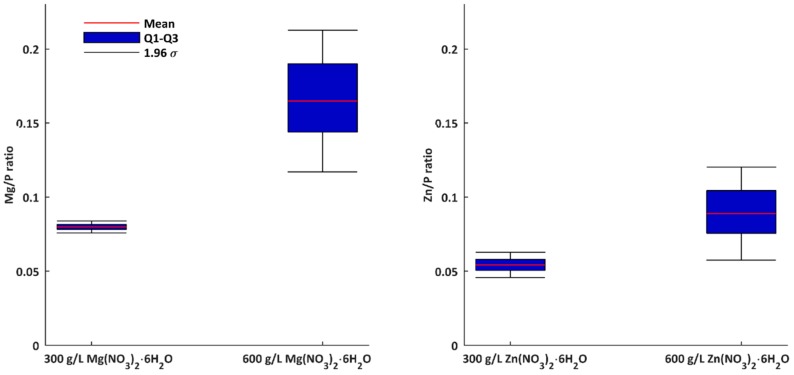
EDS results of coatings after PEO processing obtained in electrolytes composed of Mg(NO_3_)_2_·6H_2_O and Zn(NO_3_)_2_·6H_2_O in H_3_PO_4_ at concentrations of 300 g/L and 600 g/L at a voltage of 450 ± 46 V, with a pulsation frequency of 300 Hz.

In Figure 1 and Figure 2, SEM micrographs of coatings’ surfaces produced on titanium with the use of three-phase transformer with six diodes of Graetz Bridge, are presented. Some surface morphology changes, with increasing of salt concentration for both electrolytes, i.e., with Mg(NO_3_)_2_·6H_2_O or with Zn(NO_3_)_2_·6H_2_O, can be observed. This results in a better surface development from island-like structure (10 g/L of salt in electrolyte), to microporous one (300 g/L and 600 g/L of salt in electrolyte). The layers obtained in 10 g/L of magnesium-containing solution seem to be more morphologically developed than those obtained with the zinc-containing solution. Differences can be also noticed for concentrations of 300 g/L and 600 g/L, where coatings obtained in Zn(NO_3_)_2_·6H_2_O have more porous surface morphology. In addition, it should be noted that cracks on a porous coating obtained in electrolyte containing 600 g/L magnesium nitrate, are recorded. This phenomenon is very unfavorable, due to the propagation of cracks, which may lead to a faster crumble of the coating. The EDS semi-quantitative results, presented as metal-to-phosphorus ratios (Mg/P or Zn/P), have been shown in Figure 3 and Table 4. For both samples obtained in electrolytes with concentrations of 10 g/L, the EDS results indicate the presence of both magnesium or zinc and phosphorus in the studied coatings, however, due to the small signals of zinc and magnesium, qualitative analysis with the use of that method cannot be performed. The Mg/P ratio for samples obtained in the electrolyte composed of Mg(NO_3_)_2_·6H_2_O in H_3_PO_4_ for 300 g/L of that salt in the solution is equal to 0.080 ± 0.002 (first quartile 0.078; third quartile 0.082), while 600 g/L of the same salt equals 0.165 ± 0.024 (first quartile 0.140; third quartile 0.195). The Zn/P ratio for the samples obtained in electrolyte composed of Zn(NO_3_)_2_·6H_2_O in H_3_PO_4_ for 300 g/L of that salt in the solution equals 0.054 ± 0.004 (first quartile 0.050; third quartile 0.058), while for 600 g/L of the same salt, it equals 0.089 ± 0.016 (first quartile 0.075; third quartile 0.105). For both types of electrolytes, it was observed that the more salt in the electrolyte used, the higher the metal-to-phosphorus (Mg/P and Zn/P) ratios that were obtained.

**Figure 4:**
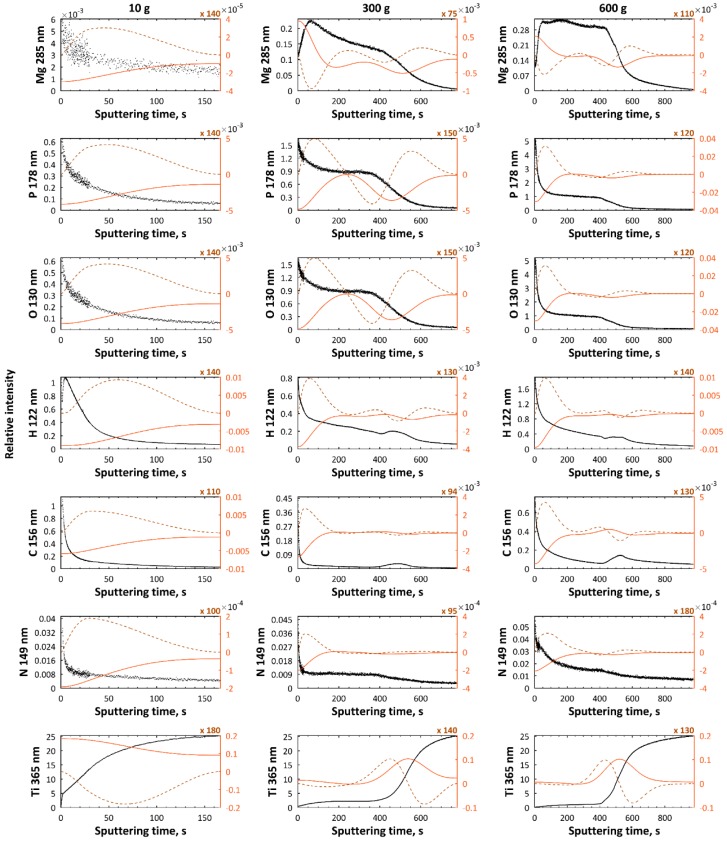
GDEOS signals (black) with first (red continuous line) and second (brown dashed line) derivatives for samples after PEO processing obtained in electrolyte composed of Mg(NO_3_)_2_·6H_2_O in H_3_PO_4_ at concentrations of 10 g/L, 300 g/L, and 600 g/L, at a voltage of 450 ± 46 V, with a pulsation frequency of 300 Hz.

**Figure 5:**
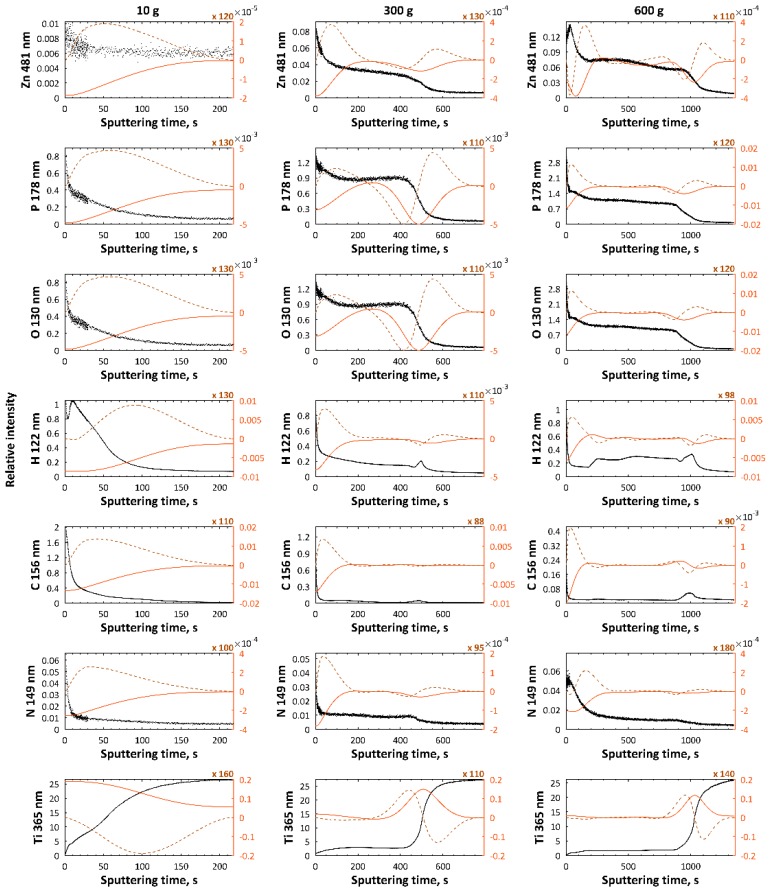
GDEOS signals (black) with first (red continuous line) and second (brown dashed line) derivatives for samples after PEO processing obtained in electrolyte composed of Zn(NO_3_)_2_·6H_2_O in H_3_PO_4_ at concentrations of 10 g/L, 300 g/L, and 600 g/L at a voltage of 450 ± 46 V, with a pulsation frequency of 300 Hz.

In Figure 4 and Figure 5, GDEOS results of PEO coatings i.e., signals of magnesium (285 nm), zinc (481 nm), phosphorus (178 nm), oxygen (130 nm), hydrogen (122 nm), carbon (156 nm), nitrogen (149 nm), and titanium (365 nm), fabricated in electrolyte, which is composed of 10 g/L, 300 g/L, and 600 g/L of Mg(NO_3_)_2_·6H_2_O or Zn(NO_3_)_2_·6H_2_O in H_3_PO_4_ at 450 ± 46 V with pulsation frequency of 300 Hz, are presented. The elements, such as magnesium, phosphorus, and oxygen, which originate from electrolyte, should be treated as the main components of the PEO coating. The titanium and magnesium signals are the smallest ones in the external top-layers, and they increase. The hydrogen and carbon signals may originate from molecules of orthophosphoric acid, water, or organic contaminations absorbed from the air or from cleaning process (alcohol molecules), while part of nitrogen signals should be interpreted as a contamination, and partly as the component of coatings, originating from nitrates of magnesium. Based on GDEOS data, the total thickness of the layers, measured as sputtering time, for magnesium- or zinc-enriched coatings, increases with increasing of Mg(NO_3_)_2_·6H_2_O or Zn(NO_3_)_2_·6H_2_O concentration from 10 g/L up to 600 g/L in H_3_PO_4_. For a concentration of 10 g/L of Mg(NO_3_)_2_·6H_2_O or Zn(NO_3_)_2_·6H_2_O, no clear sublayers of obtained PEO coatings are observed, while for samples obtained in electrolyte containing 300 g/L and 600 g/L of Mg(NO_3_)_2_·6H_2_O or Zn(NO_3_)_2_·6H_2_O, three sublayers can be distinguished as clearly visible. For samples obtained in electrolyte with 300 g/L of Mg(NO_3_)_2_·6H_2_O, the thicknesses of the first, second, and third sublayers are equal to about 50 s, 350 s, and 400 s by sputtering time, respectively, while for those treated in the solution with 600 g/L salt, the thicknesses of the first, second, and third sublayers correspond with the times of 50 s, 400 s, and 450 s, respectively. For samples obtained in the electrolyte with 300 g/L of Zn(NO_3_)_2_·6H_2_O, thicknesses of the first, second, and third sublayers are equal to about 50 s, 350 s, and 350 s by sputtering time, respectively, while for those ones treated in the solution with 600 g/L salt in it, the thicknesses of the first, second, and third sublayers correspond with the times of 150 s, 700 s, and 700 s, respectively.

**Figure 6:**
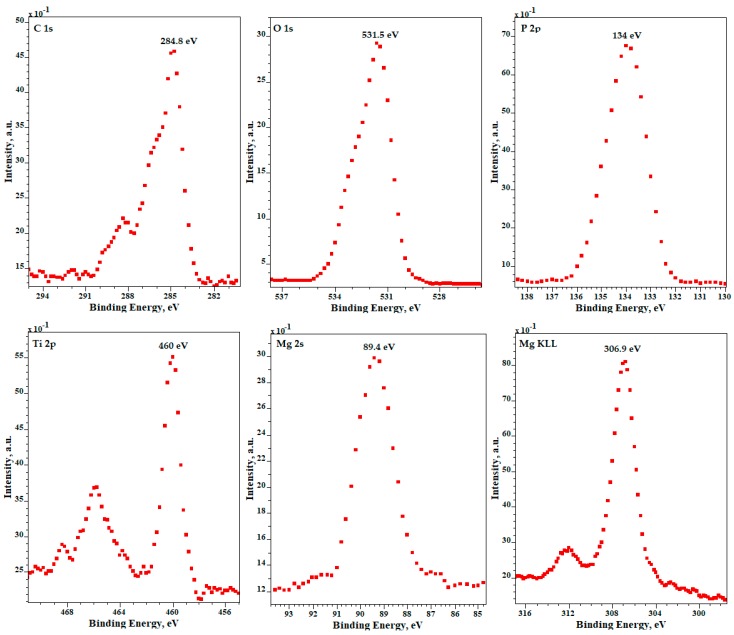
XPS results for samples after PEO processing obtained in electrolyte composed of Mg(NO_3_)_2_·6H_2_O in H_3_PO_4_ at a concentration of 600 g/L at a voltage of 450 ± 46 V, with a pulsation frequency of 300 Hz.

**Figure 7:**
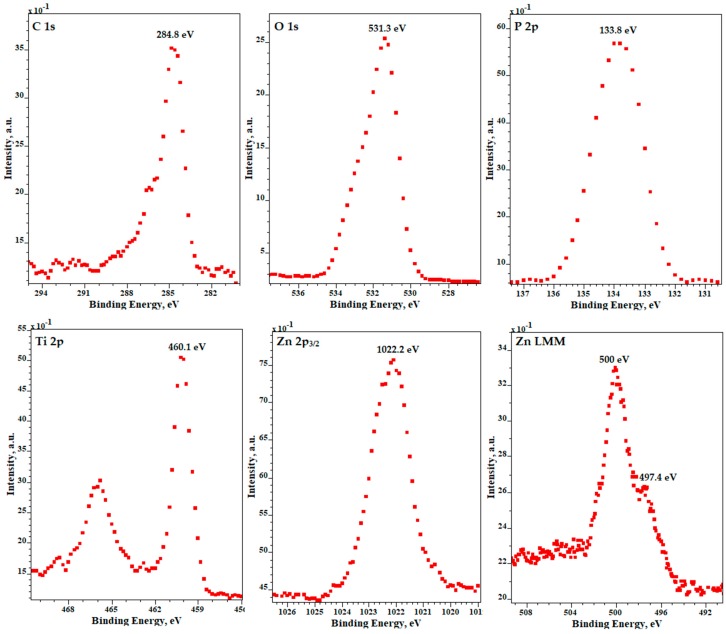
XPS results for samples after PEO processing obtained in electrolyte composed of Zn(NO_3_)_2_·6H_2_O in H_3_PO_4_ at a concentration of 600 g/L and at a voltage of 450 ± 46 V with pulsation frequency of 300 Hz.

XPS results for samples after PEO processing obtained in electrolyte composed of Mg(NO_3_)_2_·6H_2_O in H_3_PO_4_ at a concentration of 600 g/L at the voltage of 450 ± 46 V with pulsation frequency of 300 Hz are presented in Figure 6. The XPS results show that the top 10 nm layer is enriched in magnesium (Mg^2+^), phosphorus (as PO_4_^3−^, or HPO_4_^2−^, or H_2_PO_4_^−^, or P_2_O_7_^4–^), nitrogen (organic contamination), titanium (Ti^4+^), as confirmed by the binding energies of Mg 2s (89.4 eV), Mg KLL (306.9 eV), O 1s (531.5 eV), P 2p (134 eV), Ti 2p_2/3_ (460.0 eV). XPS results for samples after PEO processing obtained in electrolyte, composed of Zn(NO_3_)_2_·6H_2_O in H_3_PO_4_ at a concentration of 600 g/L at the voltage of 450 ± 46 V with pulsation frequency of 300 Hz, are presented in Figure 7. The XPS spectra show that the top 10 nm layer is enriched in zinc (Zn^2+^), phosphorus (PO_4_^3−^, or HPO_4_^2−^, or H_2_PO_4_^−^, or P_2_O_7_^4−^), and titanium (Ti^4+^), as confirmed by the binding energies of Zn 2p (1022.2 eV), Zn LMM (500 eV and 497.4 eV), O 1s (531.3 eV), P 2p (133.8 eV), Ti 2p_2/3_ (460.1 eV).

**Figure 8:**
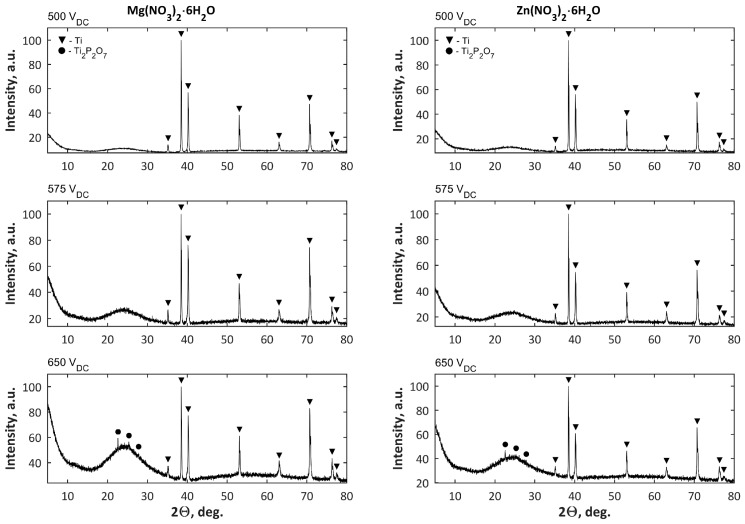
XRD results of coatings after PEO processing obtained in electrolytes composed of Mg(NO_3_)_2_·6H_2_O or Zn(NO_3_)_2_·6H_2_O in H_3_PO_4_ at a concentration of 500 g/L at three voltages, 500 V, 575 V, and 650 V.

The next step of analysis was to present the characterization of porous coatings obtained at three voltages, i.e., 500 V, 575 V, and 650 V, in electrolytes composed of Mg(NO_3_)_2_·6H_2_O in H_3_PO_4_ or Zn(NO_3_)_2_·6H_2_O in H_3_PO_4_, with the use of commercial power supply, where no voltage pulsations were recorded. A part of these results (SEM, EDS, GDOES, corrosion studies) related to characterization of these coatings were presented in reference [48]. However, for a comprehensive full analysis, the XRD analysis results should be also added, and they are presented in Figure 8. For coatings formed in both electrolytes at voltages of 500 V and 575 V, only titanium as crystalline phase (a signal from substrate) was detected. Only for samples obtained at the highest voltage, i.e., 650 V, was the Ti_2_P_2_O_7_ crystalline phase was detected. It is worth noting that the increasing of PEO voltages results in the increasing of amorphous phase in coatings’ structures.

**Figure 9:**
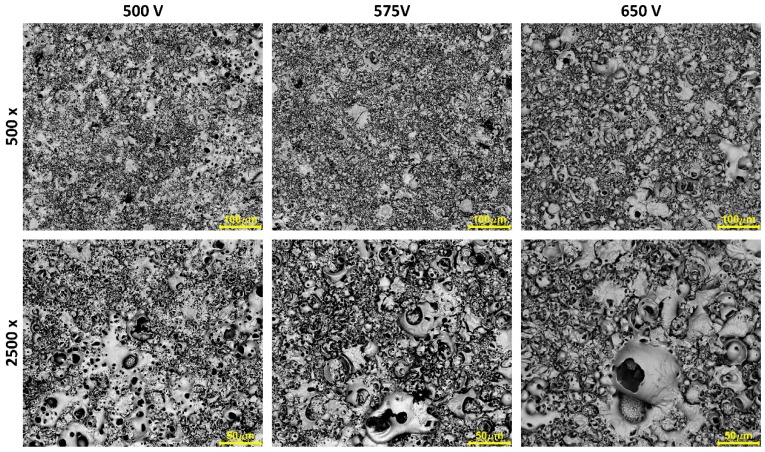

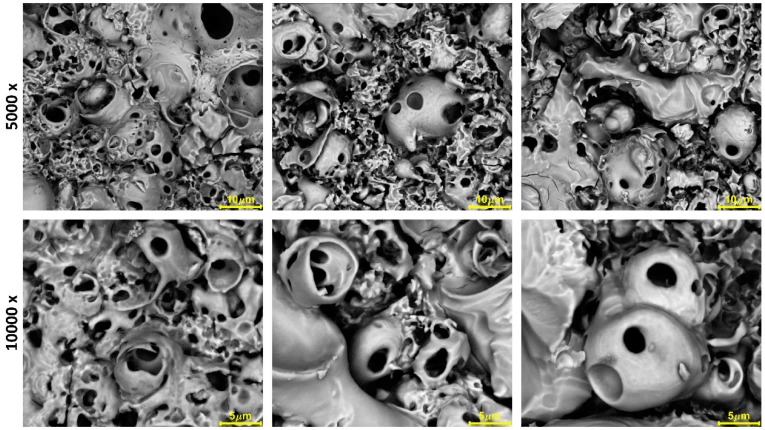
SEM micrographs of samples surfaces after PEO processing obtained in electrolytes composed of 250 g/L Mg(NO_3_)_2_·6H_2_O and 250 g/L Zn(NO_3_)_2_·6H_2_O in H_3_PO_4_ at three voltages, 500 V, 575 V and 650 V. Magnifications 500, 1000, 5000, and 10,000 times.

**Figure 10:**
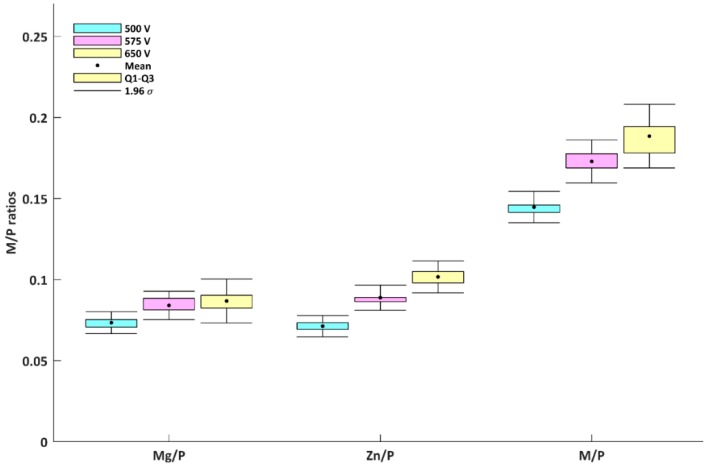
EDS results for samples after PEO processing obtained in electrolytes composed of 250 g/L Mg(NO_3_)_2_·6H_2_O and 250 g/L Zn(NO_3_)_2_·6H_2_O in H_3_PO_4_ at three voltages, 500 V, 575 V, and 650 V.

In the following part, we present the possibility of manufacturing a porous coating obtained in electrolytes composed of 250 g/L Mg(NO_3_)_2_·6H_2_O and 250 g/L Zn(NO_3_)_2_·6H_2_O in H_3_PO_4_ at the same three voltages 500 V, 575 V, 650 V, which were used in [48]. In Figure 9, the SEM micrographs of the surfaces after PEO processing, at four magnifications, are presented. The EDS results of samples obtained in electrolytes composed of 250 g/L Mg(NO_3_)_2_·6H_2_O and 250 g/L Zn(NO_3_)_2_·6H_2_O in H_3_PO_4_ as metal-to-phosphorus atomic ratios (Mg/P and Zn/P and M/P, where M = Mg + Zn), are presented in Figure 10 and Table 5. For coatings obtained at 500 V, the Mg/P, Zn/P and M/P ratios are as follows: 0.073 ± 0.003 (first quartile: 0.070, third quartile: 0.075), 0.071 ± 0.003 (first quartile: 0.069, third quartile: 0.074), 0.145 ± 0.005 (first quartile: 0.141, third quartile: 0.148), respectively. For the coatings obtained at 575 V, Mg/P, Zn/P and M/P (where M = Mg + Zn) are as follows: 0.084 ± 0.004 (first quartile: 0.081, third quartile: 0.088), 0.089 ± 0.004 (first quartile: 0.086, third quartile: 0.091), 0.173 ± 0.007 (first quartile: 0.168, third quartile: 0.178), respectively. For the coatings obtained at 650 V, Mg/P, Zn/P and M/P (where M = Mg + Zn) are as follows: 0.087 ± 0.007 (first quartile: 0.082, third quartile: 0.091), 0.102 ± 0.005 (first quartile: 0.098, third quartile: 0.106), 0.188 ± 0.010 (first quartile: 0.178, third quartile: 0.196), respectively. Both metals, i.e., magnesium and zinc, are built-in into the coating in ca. 1:1 atomic proportion, moreover, all calculated ratios show a positive correlation with applied voltage. It is also worth noting that with the increasing applied voltage, the reproducibility decreases, as indicated by calculated standard deviations.

**Figure 11:**
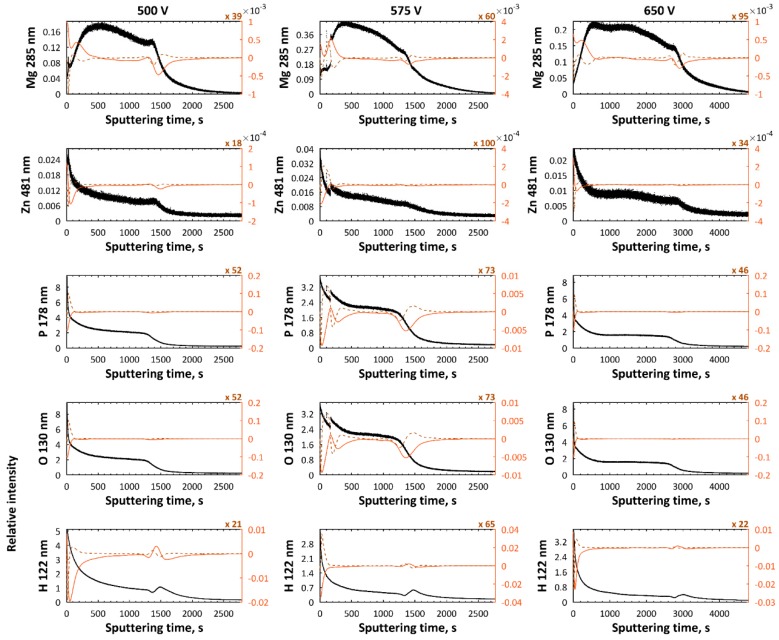

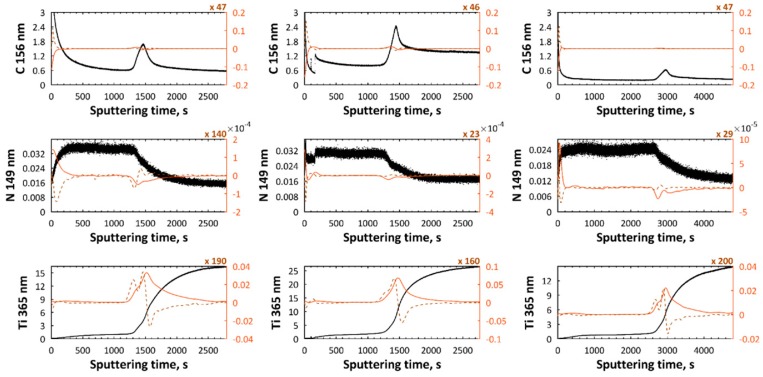
GDEOS signals (black) with first (red continuous line) and second (brown dashed line) derivatives for samples after PEO processing obtained in the electrolyte composed of 250 g/L Mg(NO_3_)_2_·6H_2_O and 250 g/L Zn(NO_3_)_2_·6H_2_O in H_3_PO_4_ at three voltages, 500 V, 575 V, and 650 V.

In Figure 11, GDEOS signals of samples and their first and second derivatives for the samples after PEO processing obtained in electrolyte composed of 250 g/L Mg(NO_3_)_2_·6H_2_O and 250 g/L Zn(NO_3_)_2_·6H_2_O in H_3_PO_4_ at three voltages, 500 V, 575 V, 650 V, are presented. The total thicknesses of layers, measured as a sputtering time, for magnesium- and zinc-enriched coatings, are equal to about 2400 s, 2400 s and 4700 s. For all three voltages, the three sublayer model is applicable. For the samples obtained at 500 V and 575 V, the thicknesses of the first, second, and third sublayers are at about 400 s, 900 s, and 1100 s, respectively. For coatings obtained at the voltage of 650 V, the thicknesses of the first, second, and third, transition sublayers are 500, 2400, and 1800 s, respectively. For all the porous castings obtained at voltages which are in the range of 500–650 V, the first sublayers are enriched in zinc, phosphorus, oxygen, hydrogen, carbon, nitrogen, and depleted in magnesium and titanium, while the second sublayers can be characterized as a semi-plateau region with non-increasing trend of all signals, except for the titanium one. In addition, the maximum for magnesium signal in that sublayer is also observed, and lower signal of zinc in comparison with the first sublayer is visible. The third, transition sublayer is characterized by an increase of titanium signal and a decrease in magnesium, zinc, phosphorus, oxygen, and nitrogen signals. The peaks in carbon and hydrogen, which originate most likely from the contamination related to the cleaning process, should be interpreted as the end of porosity of the obtained coatings.

**Figure 12:**
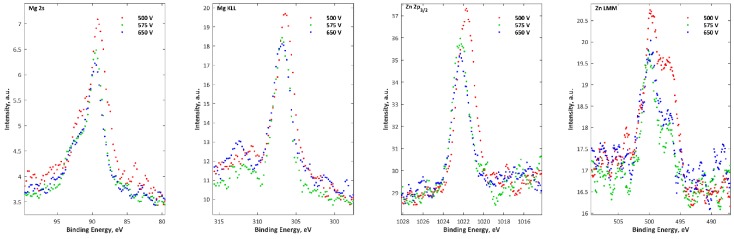

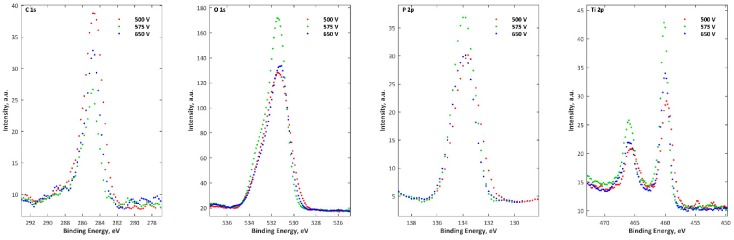
XPS signals of magnesium (Mg 2s, Mg KLL), zinc (Zn 2p_3/2_, Zn LMM) carbon (C 1s), oxygen (O 1s), phosphorus (P 2p) and titanium (Ti 2p) for samples after PEO processing obtained in electrolyte composed of 250 g/L Mg(NO_3_)_2_·6H_2_O and 250 g/L Zn(NO_3_)_2_·6H_2_O in H_3_PO_4_ at three voltages, 500 V, 575 V, and 650 V.

**Figure 13:**
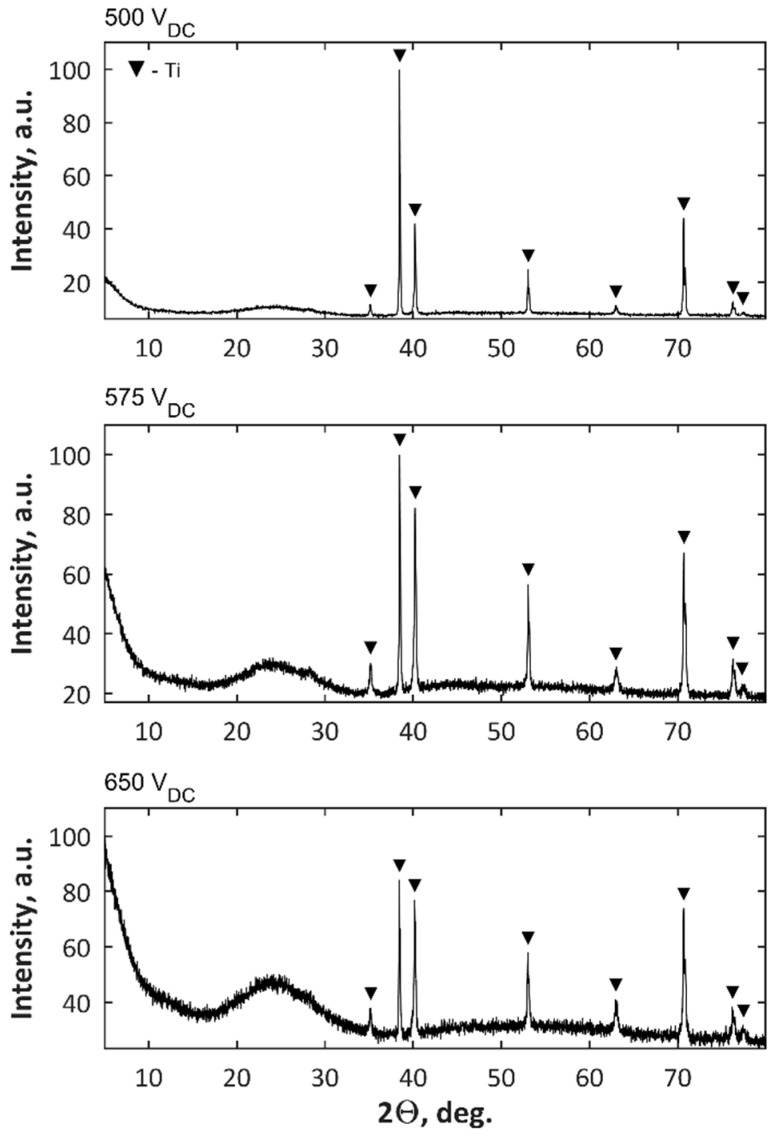
XRD results of coatings after PEO processing obtained in electrolytes composed of Mg(NO_3_)_2_·6H_2_O and Zn(NO_3_)_2_·6H_2_O in H_3_PO_4_ at a concentration of 500 g/L at three voltages 500 V, 575 V, and 650 V.

The XPS results for samples after PEO processing obtained in the electrolyte of 250 g/L Mg(NO_3_)_2_·6H_2_O and 250 g/L Zn(NO_3_)_2_·6H_2_O in 1L H_3_PO_4_ at three voltages, 500 V, 575 V, and 650 V, are presented in Figure 12. The XPS results show that the top 10 nm layer of all the obtained coatings are enriched in magnesium (Mg^2+^), zinc (Zn^2+^), phosphorus (as PO_4_^3−^, or HPO_4_^2−^, or H_2_PO_4_^−^, or P_2_O_7_^4−^), and titanium (Ti^4+^), as confirmed by the binding energies of Mg 2s (89.3–89.4 eV), Mg KLL (306.6–306.7 eV), Zn 2p (1021.9–1022.2 eV), Zn LMM (500 eV & 497 eV), O 1s (531.2–531.5 eV), P 2p (133.7–134.8 eV), Ti 2p_2/3_ (459.9–460 eV). In Figure 13, the XRD results for samples after PEO processing obtained in the electrolyte of 250 g/L Mg(NO_3_)_2_·6H_2_O and 250 g/L Zn(NO_3_)_2_·6H_2_O in 1L H_3_PO_4_ at three voltages, 500 V, 575 V, 650 V, are presented. For the coatings formed at three voltages, only titanium as a crystalline phase (a signal from matrix), alike in the case of a coating obtained in the solution with single salts at voltages of 500 V and 575 V, was detected.

## 4. Discussion

The development of technologies at the micro scale provides the opportunity to increase applications of micromachines, most often in medicine. In the available literature, it is possible to find out some examples, such as multiplexed microfluidic platform for bone marker measurement [56] or integrated microfluidic devices, e.g., for DNA analysis, cell handling, sorting, and general analysis [57]. It should be pointed out that the main novelty of this work is fabrication and characterization of new porous coatings enriched in magnesium and zinc in new electrolytes, based on concentrated phosphoric acid, with the addition of magnesium and zinc nitrates, which may be used as biocompatible sensors’ housing. They may be put into the bone to monitor, for instance, the amount of bacteria in wounds. In addition, porous housing may be used, e.g., as a drug delivery polymer system. The preliminary studies have turned the attention on the possibility to create porous coatings in electrolytes containing single magnesium or zinc nitrate, as well as in those with two salts. It was found that the coatings obtained in 10 g/L of magnesium-containing solution are characteristic, with more developed surface than those ones formed in electrolyte with the same amount of zinc nitrate. However, the truly porous coatings have been obtained for the salt contents in a solution of 300 and 600 g/L, respectively. The other critical issues of some obtained surfaces are coatings’ cracking, which has been visible especially on these samples formed in electrolyte containing 600 g/L magnesium nitrate. That case is very unfavorable due to the propagation of cracks, which may lead to faster coat crumbling, e.g., during exploitation. Based on the EDS results of Mg/P and Zn/P ratios, it was concluded that the building-in of the magnesium ions into the phosphate structure is more probable than the zinc ones. Generally, it should be noted that the more total amount of salts in electrolyte, the higher the metal-to-phosphorus ratios in coatings that are observed. The XPS studies, which complement the information on the chemical composition of the 10 nm depth coating, allowed it to be revealed that the external (top) coatings’ part is composed mainly of magnesium (Mg^2+^), (Zn^2+^), titanium (Ti^4+^), and phosphorous (PO_4_^3−^, or HPO_4_^2−^, or H_2_PO_4_^−^, or P_2_O_7_^4−^). The depth profiles, which were performed by GDOES, have clearly displayed that for the concentration of 10 g/L of Mg(NO_3_)_2_·6H_2_O or Zn(NO_3_)_2_·6H_2_O in electrolytes, no clear sublayers of the obtained PEO coatings were observed, while for samples obtained in the electrolyte with 300 g/L and 600 g/L of the same salts, three sublayers could be detected. It was also observed that increasing the amount of salt in electrolyte solution results in the formation of thicker coatings, while the increasing of PEO voltage, for the same electrolyte, results in growing the amorphous phase, as well as increasing the Zn/P and Mg/P ratios.

## 5. Conclusions

(a) The more salt (Mg(NO_3_)_2_·6H_2_O and Zn(NO_3_)_2_·6H_2_O) in electrolyte, the higher the metal-to-phosphorus (Mg/P and Zn/P) ratios that are obtained.
(b) The more salt (Mg(NO_3_)_2_·6H_2_O and/or Zn(NO_3_)_2_·6H_2_O) in electrolyte, the thicker the coating that is formed.
(c) The increase of PEO voltages results in the increase of amorphous phase in the coatings’ structures.
(d) The higher voltage of PEO treatment, the higher are Zn/P and Mg/P ratios in coatings obtained in the electrolytes containing Mg(NO_3_)_2_·6H_2_O and 250 g/L Zn(NO_3_)_2_·6H_2_O.
(e) The top 10 nm layers of the studied coatings are composed of magnesium (Mg^2+^), zinc (Zn^2+^), phosphorous (PO_4_^3−^, or HPO_4_^2−^, or H_2_PO_4_^−^, or P_2_O_7_^4−^), and titanium (Ti^4+^).

## Figures and Tables

**Table 1:** Examples of aqueous electrolytes used in plasma electrolytic oxidation (PEO) treatment with voltage conditions.

Electrolytes	Voltages	Ref.
Na_2_CO_3_ and Na_3_PO_4_, and (CH_3_COO)_2_Ca·H_2_O	200–500 V (900 Hz)	[33]
(CH_3_COO)_2_Ca·H_2_O and C_3_H_7_Na_2_O_6_P·5H_2_O		
Na_4_P_2_O_7_·10H_2_O and KOH, NaAlO_2_	0–300 V	[34]
Na_2_B_4_O_7_·10H_2_O and (CH_3_COO)_2_Mn·4H_2_O	450–500 V	[35]
(CH_3_COO)_2_Ca·H_2_O	230 V	[36]
(CH_3_COO)_2_Ca·H_2_O and NaH_2_PO_4_·2H_2_O	260–420 V	[37]
NH_4_H_2_PO_4_, CaCl_2_, NaH_2_PO_4_, (CH_3_COO)_2_Ca	0–500 V	[38]
KOH	290 V (100–200 Hz)	[39]
KOH	350 V (1000 Hz)	[40]
(NaPO_3_)_6_, NaF and NaAlO_2_	150–200 V	[41]
K_2_Al_2_O_4_, Na_3_PO_4_, NaOH	400 V	[42]
CaCl_2_ and KH_2_PO_4_	320–340 V	[43]
H_2_SO_4_ and Ti_2_(SO_4_)_3_	1100 V	[44]
Na_2_(EDTA) and CaO, Ca(H_2_PO_4_)_2_ and Na_2_SiO_3_·H_2_O	350 V (200 Hz)	[45]
Na_2_SiO_3_, and NaOH	280 V	[46]

**Table 2:** Electrolytes used to oxidize the titanium by (PEO) treatment.

Power Supply	Voltage (V)	Salt	Salt Concentration (g/L)
Three-phase transformer with Graetz Bridge	450 ± 46	Mg(NO_3_)·6H_2_O	10
			300
			600
		Zn(NO_3_)_2_·6H_2_O	10
			300
			600
PWR 1600 H, Multi Range DC Power Supply	500	Mg(NO_3_)_2_·6H_2_O	500
	575		
	650		
	500	Zn(NO_3)2_·6H_2_O	500
	575		
	650		
	500	Mg(NO_3_)_2_·6H_2_O & Zn(NO_3_)_2_·6H_2_O	250 + 250
	575		
	650		

**Table 3:** Set-up descriptions of SEM, EDS, XPS, GDEOS, and XRD equipment.

SEM & EDS	XPS	GDOES	XRD
Quanta 250 & 650 FEG (SEM: Field Electron and Iron Company, Hillsboro, OR, USA EDS: Thermo Fisher Scientific, Madison, WI, USA)	SCIENCE SES 2002 (SCIENTA AB, ScientaOmicron, Uppsala, Sweden)	Horiba Scientific GD Profiler 2 (HORIBA Scientific, Palaiseau, France)	Bruker-AXS D8 Advance (BRUKER Corporation, Billerica, MA, USA)
High Vacuum	monochromatic (Gammadata-Scienta) Al K(alpha) X-ray source	radio frequency (RF) pulsed source	2Θ/Θ geometry
ESEM mode	(hν = 1486.6 eV) (18.7 mA, 13.02 kV)	pressure: 700 Pa, power: 40 W	radiation CuKα Ni filter
EDS Noran System Six	energy step 0.2 eV	frequency: 3000 Hz, duty cycle: 0.25	voltage 40 kV current 40 mA
ETD & BSED detectors	step time 200 ms	anode diameter: 4 mm	step by step mode of 0.014 2Θ with an interval of 0.25 s per step

**Table 4:** Statistical description of Mg/P and Zn/P ratios based on atomic percent.

Salt	Concentration	Mean	Stand. Dev.	First Quartile	Third Quartile
Mg(NO_3_)_2_·6H_2_O	300 g/L	0.080	0.002	0.078	0.082
	600 g/L	0.165	0.024	0.140	0.195
Zn(NO_3_)_2_·6H_2_O	300 g/L	0.054	0.004	0.050	0.058
	600 g/L	0.089	0.016	0.075	0.105

**Table 5:** Statistical description of Mg/P, Zn/P and M/P ratios based on atomic percent (M = Mg + Zn).

Ratios	Voltage	Mean	Stand. Dev.	First Quartile	Third Quartile
Mg/P	500 V	0.073	0.003	0.070	0.075
	575 V	0.084	0.004	0.081	0.088
	650 V	0.087	0.007	0.082	0.091
Zn/P	500 V	0.071	0.003	0.069	0.074
	575 V	0.089	0.004	0.086	0.091
	650 V	0.102	0.005	0.098	0.106
M/P	500 V	0.145	0.005	0.141	0.148
	575 V	0.173	0.007	0.168	0.178
	650 V	0.188	0.010	0.178	0.196

## References

[1] Stojadinović S., Jovović J., Petković M., Vasilić R., Konjević N. (2011). Spectroscopic and real-time imaging investigation of tantalum plasma electrolytic oxidation (PEO). Surf. Coat. Technol..

[2] Fidan S., Muhaffel F., Riool M., Cempura G., de Boer L., Zaat S.A.J., Czyrska-Filemonowicz A., Cimenoglu H. (2017). Fabrication of oxide layer on zirconium by micro-arc oxidation: Structural and antimicrobial characteristics. Mater. Sci. Eng. C.

[3] Aktug S.L., Kutbay I., Usta M. (2017). Characterization and formation of bioactive hydroxyapatite coating on commercially pure zirconium by micro arc oxidation. J. Alloys Compd..

[4] Stojadinović S., Tadić N., Ćirić A., Vasilić R. (2018). Photoluminescence properties of Eu^3+^ doped HfO_2_ coatings formed by plasma electrolytic oxidation of hafnium. Opt. Mater..

[5] Stojadinović S., Tadić N., Vasilić R. (2017). Plasma electrolytic oxidation of hafnium. Int. J. Refract. Met. Hard Mater..

[6] Sowa M., Simka W. (2018). Electrochemical behavior of plasma electrolytically oxidized niobium in simulated physiological environment. Surf. Coat. Technol..

[7] Sowa M., Worek J., Dercz G., Korotin D.M., Kukharenko A.I., Kurmaev E.Z., Cholakh S.O., Basiaga M., Simka W. (2016). Surface characterisation and corrosion behaviour of niobium treated in a Ca- and P-containing solution under sparking conditions. Electrochim. Acta.

[8] Sowa M., Woszczak M., Kazek-Kęsik A., Dercz G., Korotin D.M., Zhidkov I.S., Kurmaev E.Z., Cholakh S.O., Basiaga M., Simka W. (2017). Influence of process parameters on plasma electrolytic surface treatment of tantalum for biomedical applications. Appl. Surf. Sci..

[9] Sowa M., Kazek-Kȩsik A., Socha R.P., Dercz G., Michalska J., Simka W. (2013). Modification of tantalum surface via plasma electrolytic oxidation in silicate solutions. Electrochim. Acta.

[10] Farhadi S.S., Aliofkhazraei M., Barati Darband G., Abolhasani A., Sabour Rouhaghdam A. (2017). Corrosion and wettability of PEO coatings on magnesium by addition of potassium stearate. J. Magnes. Alloys.

[11] Egorkin V.S., Gnedenkov S.V., Sinebryukhov S.L., Vyaliy I.E., Gnedenkov A.S., Chizhikov R.G. (2018). Increasing thickness and protective properties of PEO-coatings on aluminum alloy. Surf. Coat. Technol..

[12] Mingo B., Arrabal R., Mohedano M., Llamazares Y., Matykina E., Yerokhin A., Pardo A. (2018). Influence of sealing post-treatments on the corrosion resistance of PEO coated AZ91 magnesium alloy. Appl. Surf. Sci..

[13] Gao Y., Yerokhin A., Matthews A. (2015). Mechanical behaviour of cp-magnesium with duplex hydroxyapatite and PEO coatings. Mater. Sci. Eng. C.

[14] Kasalica B., Radić-Perić J., Perić M., Petković-Benazzouz M., Belča I., Sarvan M. (2016). The mechanism of evolution of microdischarges at the beginning of the PEO process on aluminum. Surf. Coat. Technol..

[15] Guan Y., Xia Y., Li G. (2008). Growth mechanism and corrosion behavior of ceramic coatings on aluminum produced by autocontrol AC pulse PEO. Surf. Coat. Technol..

[16] Hryniewicz T., Rokosz K., Sandim H.R.Z. (2012). SEM/EDX and XPS studies of niobium after electropolishing. Appl. Surf. Sci..

[17] Hryniewicz T., Rokosz K., Rokicki R., Prima F. (2015). Nanoindentation and XPS studies of Titanium TNZ alloy after electrochemical polishing in a magnetic field. Materials.

[18] Rokosz K., Lahtinen J., Hryniewicz T., Rzadkiewicz S. (2015). XPS depth profiling analysis of passive surface layers formed on austenitic AISI 304L and AISI 316L SS after high-current-density electropolishing. Surf. Coat. Technol..

[19] Yao Z., Cui R., Jiang Z., Wang F. (2007). Effects of duty ratio at low frequency on growth mechanism of micro-plasma oxidation ceramic coatings on Ti alloy. Appl. Surf. Sci..

[20] Curran J.A., Kalkanci H., Magurova Y., Clyne T.W. (2007). Mullite-rich plasma electrolytic oxide coatings for thermal barrier applications. Surf. Coat. Technol..

[21] Lukiyanchuk I.V., Chernykh I.V., Rudnev V.S., Ustinov A.Y., Tyrina L.M., Nedozorov P.M., Dmitrieva E.E. (2014). Catalytically active cobalt-copper-oxide layers on aluminum and titanium. Prot. Metals Phys. Chem. Surf..

[22] Hu H., Zhang W., Qiao Y., Jiang X., Liu X., Ding C. (2012). Antibacterial activity and increased bone marrow stem cell functions of Zn-incorporated TiO_2_ coatings on titanium. Acta Biomater..

[23] Peng B.Y., Nie X., Chen Y. (2014). Effects of surface coating preparation and sliding modes on titanium oxide coated titanium alloy for aerospace applications. Int. J. Aerosp. Eng..

[24] El Achhaba M., Schierbaum K. (2012). Structure and hydrogen sensing properties of plasma electrochemically oxidized titanium foils. Procedia Eng..

[25] Tekin K.C., Malayoglu U., Shrestha S. (2016). Tribological behaviour of plasma electrolytic oxide coatings on Ti6Al4V and cp-Ti alloys. Surf. Eng..

[26] Han Y., Hong S.H., Xu K., Cheng S., Feng W., Li B., Wang Y., Jia D., Zhou Y., Göttlicher M. (2002). Synthesis of nanocrystalline titania films by micro-arc oxidation. Mater. Lett..

[27] Han Y., Xu K. (2004). Photoexcited formation of bone apatite-like coatings on micro-arc oxidized titanium. J. Biomed. Mater. Res..

[28] Huang P., Xu K.W., Han Y., Luo Q., Zhang D.Q., Li X.W., Zhao X., Sun W., Zhou Y., Göttlicher M. (2005). Preparation and apatite layer formation of plasma electrolytic oxidation film on titanium for biomedical application. Mater. Lett..

[29] Song W.H., Jun Y.K., Han Y., Hong S.H., Kim H.E., Heo S.J., Koak J.Y. (2004). Biomimetic apatite coatings on micro-arc oxidized titania. Biomaterials.

[30] Zhang Y.M., Bataillon-Linez P., Huang P., Zhao Y.M., Han Y., Traisnel M., Xu K.W., Hildebrand H.F. (2003). Surface analyses of micro-arc oxidized and hydrothermally treated titanium and effect on osteoblast behavior. J. Biomed. Mater. Res..

[31] Li L.H., Kong Y.M., Kim H.W., Kim Y.W., Kim H.E., Heo S.J., Koak J.Y. (2004). Improved biological performance of Ti implants due to surface modification by micro-arc oxidation. Biomaterials.

[32] Lee S.H., Kim H.W., Lee E.J., Li L.H., Kim H.E. (2006). Hydroxyapatite–TiO_2_ hybrid coating on Ti implants. J. Biomater. Appl..

[33] Han Y., Hong S.H., Xu K., Puz’ A.V., Gnedenkov A.S., Minaev A.N., He J.L., Jia D., Zhou Y., Göttlicher M. (2003). Structure and in vitro bioactivity of titania-based films by micro-arc oxidation. Surf. Coat. Technol..

[34] Teh T.H., Berkani A., Mato S., Skeldon P., Thompson G.E., Habazaki H., Shimizu K., Habazaki H., Zhou Y., Göttlicher M. (2003). Initial stages of plasma electrolytic oxidation of titanium. Corros. Sci..

[35] Rudnev V.S., Vasilyeva M.S., Kondrikov N.B., Tyrina L.M., Feng J., Wang Y.J., Wu K., Habazaki H., Zhou Y., Göttlicher M. (2005). Plasma-electrolytic formation, composition and catalytic activity of manganese oxide containing structures on titanium. Appl. Surf. Sci..

[36] Ryu H.S., Song W.H., Hong S.H., Nedozorov P.M., Kondrikov N.B., Didenko N.A., Gerasimenko A.V., Habazaki H., Zhou Y., Göttlicher M. (2005). Biomimetic apatite induction on Ca-containing titania. Curr. Appl. Phys..

[37] Chen J.Z., Shi Y.L., Wang L., Yan F.Y., Zhang F.Y., Janghorban K., Wang Y., Jia D., Zhou Y., Göttlicher M. (2006). Preparation and properties of hydroxyapatite-containing titania coating by micro-arc oxidation. Mater. Lett..

[38] Matykina E., Montuori M., Gough J., Monfort F., Berkani A., Skeldon P., Thompson G.E., Habazaki H., Zhou Y., Göttlicher M. (2006). Spark anodising of titanium for biomedical applications. Trans. IMF.

[39] Han I.H., Choi J.H., Zhao B.H., Baik H.K., Lee I.S., Minaev A.N., He J.L., Jia D., Zhou Y., Göttlicher M. (2006). Effects of electrical wave form on pore size of micro-arc oxidized TiO_2_ film. Key Eng. Mater..

[40] Shokouhfar M., Dehghanian C., Montazeri M., Baradaran A., Avramenko V.A., Tsvetnikov A.K., Sergienko V.I., Kurjavyj V.G., Ye H., Opra D.P. (2012). Preparation of ceramic coating on Ti substrate by plasma electrolytic oxidation in different electrolytes and evaluation of its corrosion resistance: Part II. Appl. Surf. Sci..

[41] Zhu L., Ye X., Tang G., Zhao N., Gong Y., Zhao Y., Zhao J., Zhang X. (2006). Corrosion test, cell behavior test, and in vivo study of gradient TiO_2_ layers produced by compound electrochemical oxidation. J. Biomed. Mater. Res. A.

[42] Habazaki H., Onodera T., Fushimi K., Konno H., Toyotake K., Zhao Y., Zhao J., Zhang X., Liang Z.H., Landers R. (2007). Spark anodizing of β-Ti alloy for wear-resistant coating. Surf. Coat. Technol..

[43] Kim M.S., Ryu J.J., Sung Y.M., Nan K., Han Y., Ustinov A.Y., He J.L., Chu P.K., Matykina E., Landers R. (2007). One-step approach for nano-crystalline hydroxyapatite coating on titanium via micro-arc oxidation. Electrochem. Commun..

[44] Ragalevičius R., Stalnionis G., Niaura G., Jagminas A. (2008). Micro-arc oxidation of Ti in a solution of sulfuric acid and Ti^+3^ salt. Appl. Surf. Sci..

[45] Zhang W., Du K., Yan C., Wang F., Chuvilin A., Jiang J.Z., Valiev R.Z., Qi M., Fecht H.J., Göttlicher M. (2008). Preparation and characterization of a novel Si-incorporated ceramic film on pure titanium by plasma electrolytic oxidation. Appl. Surf. Sci..

[46] Lebukhova N.V., Rudnev V.S., Kirichenko E.A., Chigrin P.G., Lukiyanchuk I.V., Yarovaya T.P., Zavidnaya A.G., Puz’ A.V., Khlusov I.A., Opra D.P. (2016). Effect of the structure of the oxidized titanium surface on the particle size and properties of the deposited copper-molybdate catalyst. Prot. Met. Phys. Chem. Surf..

[47] Rokosz K., Hryniewicz T., Gaiaschi S., Chapon P., Raaen S., Pietrzak K., Malorny W. (2017). Characterisation of calcium- and phosphorus-enriched porous coatings on cp titanium grade 2 fabricated by plasma electrolytic oxidation. Metals.

[48] Rokosz K., Hryniewicz T., Gaiaschi S., Chapon P., Raaen S., Pietrzak K., Malorny W., Salvador Fernandes J. (2018). Characterization of porous phosphate coatings enriched with magnesium or zinc on cp titanium grade 2 under DC plasma electrolytic oxidation. Metals.

[49] Rokosz K., Hryniewicz T., Raaen S. (2016). Development of plasma electrolytic oxidation for improved Ti6Al4V biomaterial surface properties. Int. J. Adv. Manuf. Technol..

[50] Rokosz K., Hryniewicz T., Raaen S., Chapon P. (2016). Investigation of porous coatings obtained on Ti-Nb-Zr-Sn alloy biomaterial by plasma electrolytic oxidation: Characterisation and modelling. Int. J. Adv. Manuf. Technol..

[51] Nelis T., Payling R., Barnett N.W. (2002). Practical guide to glow discharge optical emission spectroscopy. RSC Analytical Spectroscopy Monographs.

[52] Casa Software Ltd. (2009). CasaXPS: Processing software for XPS, AES, SIMS and More. http://www.casaxps.com.

[53] Rokosz K., Hryniewicz T., Raaen S. (2014). Cr/Fe ratio by XPS spectra of magnetoelectropolished AISI 316L SS fitted by Gaussian-Lorentzian shape lines. Teh. Vjesn..

[54] Naumkin A.V., Kraut-Vass A., Gaarenstroom S.W., Powell C.J. (2012). NIST X-ray Photoelectron Spectroscopy Database: NIST Standard Reference Database 20, Version 4.1. https://srdata.nist.gov/xps/.

[55] Moulder J.F., Stickle W.F., Sobol P.E., Bomben K.D., Chastain J. (1992). Handbook of X-ray Photoelectron Spectroscopy.

[56] Khashayar P., Amoabediny G., Larijani B., Hosseini M., Verplancke R., Schaubroeck D., Van Put S., Razi F., De Keersmaecker M., Adriaens A. (2017). A multiplexed microfluidic platform for bone marker measurement: A proof-of-concept. Micromachines.

[57] Erickson D., Li D. (2004). Integrated microfluidic devices. Anal. Chim. Acta.

